# Genomic landscape in acute myeloid leukemia and its implications in risk classification and targeted therapies

**DOI:** 10.1186/s12929-020-00674-7

**Published:** 2020-07-21

**Authors:** Hsin-An Hou, Hwei-Fang Tien

**Affiliations:** Division of Hematology, Department of Internal Medicine, National Taiwan University Hospital, National Taiwan University, Taipei, Taiwan

**Keywords:** Acute myeloid leukemia, Genetic markers, Risk stratification, Therapeutics

## Abstract

Acute myeloid leukemia (AML) is a heterogeneous hematologic malignancy in terms of clinical features, underlying pathogenesis and treatment outcomes. Recent advances in genomic techniques have unraveled the molecular complexity of AML leukemogenesis, which in turn have led to refinement of risk stratification and personalized therapeutic strategies for patients with AML. Incorporation of prognostic and druggable genetic biomarkers into clinical practice to guide patient-specific treatment is going to be the mainstay in AML therapeutics. Since 2017 there has been an explosion of novel treatment options to tailor personalized therapy for AML patients. In the past 3 years, the U.S. Food and Drug Administration approved a total of eight drugs for the treatment of AML; most specifically target certain gene mutations, biological pathways, or surface antigen. These novel agents are especially beneficial for older patients or those with comorbidities, in whom the treatment choice is limited and the clinical outcome is very poor. How to balance efficacy and toxicity to further improve patient outcome is clinically relevant. In this review article, we give an overview of the most relevant genetic markers in AML with special focus on the therapeutic implications of these aberrations.

## Introduction

Acute myeloid leukemia (AML) is a clonal hematologic malignancy with great variability in the clinical features, pathogenesis and treatment outcomes [1, 2]. The incidence of AML is increasing over time and males are more prone to develop AML than female [3]. It is the most common form of acute leukemia in adults and accounts for the highest percentage of leukemia death [4].

Although the majority of fit patients initially achieve complete remission (CR) after induction chemotherapy, a significant number of patients eventually experience disease relapse or refractoriness [5, 6], which underscores the unmet need for novel therapies. Until recently, the treatment options for AML have been limited to cytotoxic chemotherapy and allogeneic hematopoietic stem cell transplantation (HSCT). The combination of an anthracycline and cytarabine, widely known as the “7 + 3” regimen, has been the cornerstone of induction therapy for AML for decades. Efforts to improve the response rate and overall survival (OS) had previously focused on dose intensification of cytarabine and the addition of pharmaceutically distinct agents to induction and the following consolidation chemotherapy [7–10]. Nevertheless, the clinical outcome of AML patients treated with these cytotoxic drugs, even in combination with HSCT, is not satisfactory. The long-term survival in de novo patients younger than 60 years is approximately 30–50%, and that in older patients and those with secondary AML is less than 10% [2, 11, 12], highlighting the urgent need for novel treatment to improve the survival. Herein we give an overview of the most relevant genetic markers in AML and their clinical implications in risk-stratification and targeted therapy.

### Changes of AML classification over time with the advance of genomics

AML was initially classified by the French-American-British (FAB) Cooperative Group in 1976 according to the cell lineage of leukemic cells and the extent of their differentiation based on the cell morphology and cytochemical staining of bone marrow (BM) cells [13]. However, it was far from perfect to precisely stratify this heterogeneous disease and predict outcome. The identification of recurrent cytogenetic abnormalities advance our understanding of the AML biology and drive decision-making in clinical practice [14–16]. In 2001, the World Health Organization (WHO) introduced a new classification system by including recurrent cytogenetic abnormalities as criteria [17], which was followed by a revised version in 2008 [18].

Advances in genomic techniques and research have greatly shed light on our understanding of cancer biology. It is found that more than 95% of AML patients have driving and co-concurring mutations regardless of the presence of cytogenetic abnormalities [19–21]. Because of the importance of genetic aberrations, AML with recurrent genetic abnormalities (either cytogenetic or molecular genetic) is classified as the first subtype of AML, together with five other subtypes, in the latest 2016 WHO Classification (Table 1) [1].

**Table 1:** The 2016 WHO classification of acute myeloid leukemia (AML) and related neoplasms

**AML with recurrent genetic abnormalities**	
AML with t(8;21)(q22;q22.1);RUNX1-RUNX1T1	
AML with inv. (16)(p13.1q22) or t(16;16)(p13.1;q22);CBFB-MYH11	
APL with PML-RARA	
AML with t(9;11)(p21.3;q23.3);MLLT3-KMT2A	
AML with t(6;9)(p23;q34.1);DEK-NUP214	
AML with inv. (3)(q21.3q26.2) or t(3;3)(q21.3;q26.2); GATA2, MECOM	
AML (megakaryoblastic) with t(1;22)(p13.3;q13.3);RBM15-MKL1	
Provisional entity: AML with BCR-ABL1	
AML with mutated NPM1	
AML with biallelic mutations of CEBPA	
Provisional entity: AML with mutated RUNX1	
**AML with myelodysplasia-related changes**	
**Therapy-related myeloid neoplasms**	
**AML, NOS**	
AML with minimal differentiation	
AML without maturation	
AML with maturation	
Acute myelomonocytic leukemia	
Acute monoblastic/monocytic leukemia	
Pure erythroid leukemia	
Acute megakaryoblastic leukemia	
Acute basophilic leukemia	
Acute panmyelosis with myelofibrosis	
**Myeloid sarcoma**	
**Myeloid proliferations related to Down syndrome**	
Transient abnormal myelopoiesis (TAM)	
Myeloid leukemia associated with Down syndrome	

APL, acute promyelocytic leukemia; NOS, not otherwise specified

### Genomic landscape in AML and its implication in risk classification

**Fig. 1:**
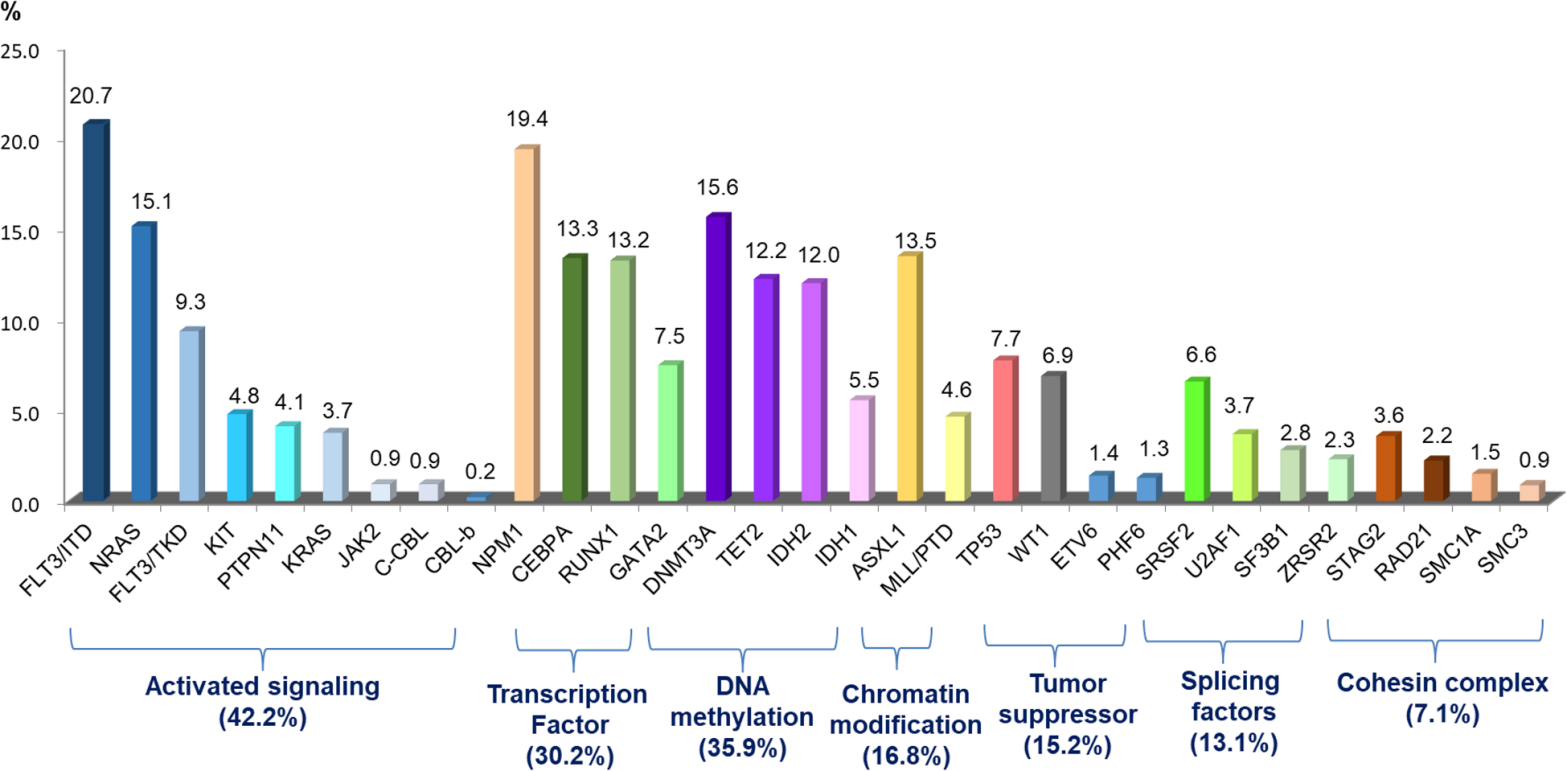
Common molecular gene mutations and their incidences in 763 AML patients in Taiwan. The data are derived from the mutation analyses of 763 patients diagnosed and treated at the National Taiwan University Hospital

**Fig. 2:**
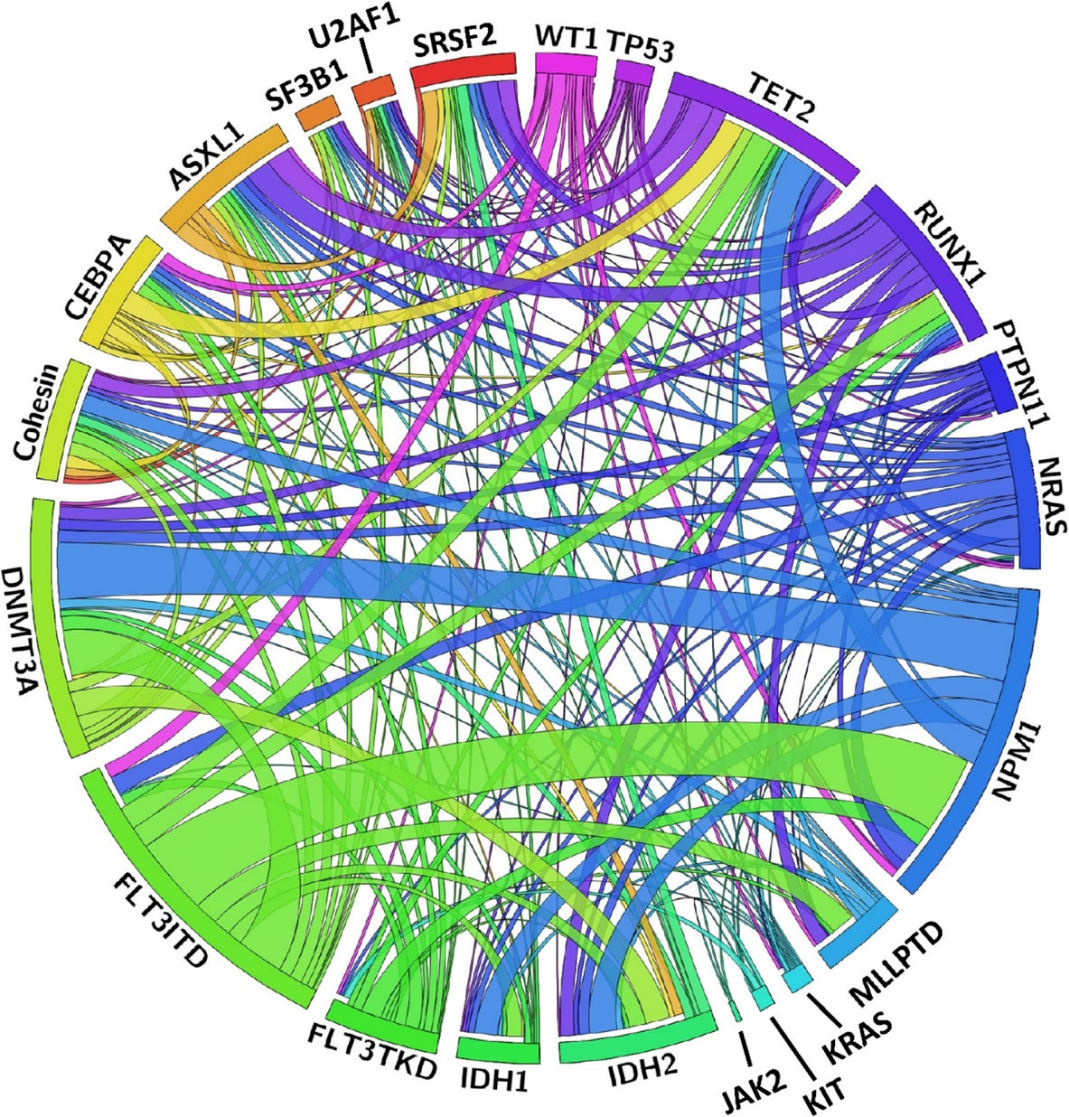
The Circos plots depicting the relative frequency and pairwise co-occurrence of genetic alterations in 500 AML patients in Taiwan. The length of the arc corresponds to the frequency of the first gene mutation, and the width of the ribbon corresponds to the proportion of the second gene mutation. The data are derived from the mutation analyses of 500 patients diagnosed and treated at the National Taiwan University Hospital

**Fig. 3:**
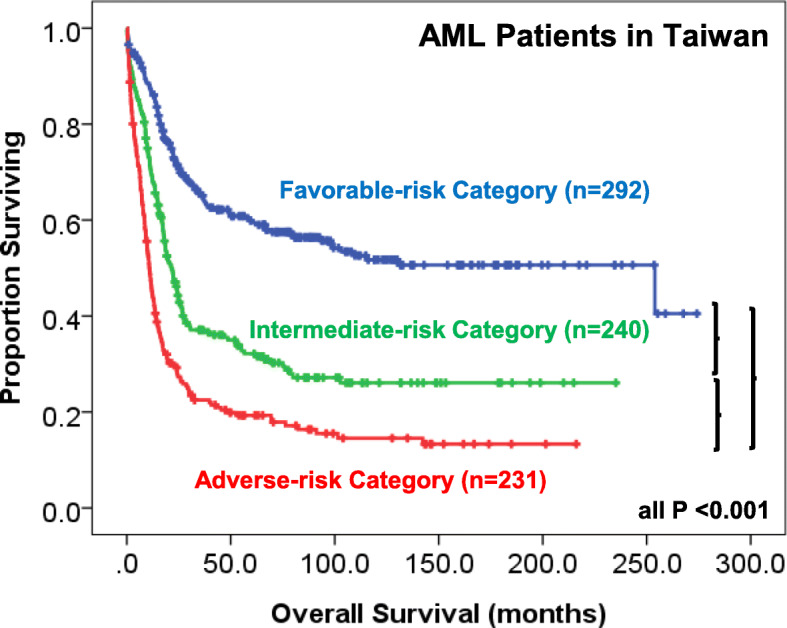
Survival curves of 763 AML patients in Taiwan stratified according to the 2017 ELN risk stratification. The data are derived from the mutation analyses of 763 patients diagnosed and treated at the National Taiwan University Hospital

The mutations that have a putative role in AML pathogenesis are classified into eight categories according to their biological function, including those involving myeloid transcription-factor genes, *NPM1*, tumor suppressors, signaling genes, DNA methylation, chromatin modifier, cohesin complex and splicing factors (Table 2) [2, 19, 21]. The incidences of common molecular mutations in our AML cohort is shown in Fig. 1. It’s common that more than one mutations occur concurrently in the same patient (Fig. 2) indicating a role of concerted interaction of mutations in the pathogenesis of AML [22, 23]. The discovery of molecular genetic alterations has led to the refinement of prognostication in AML. The 2017 European LeukemiaNet (ELN) recommendation for risk-stratification of AML [24] (Table 3) is the most widely used model in current clinical practice since it incorporates cytogenetic changes and gene mutation status, including *FLT3-*ITD allelic ratio, into the risk classification which largely enhances the stratification power compared with the 2010 version of the ELN recommendations [25]. Based on integrated analysis of clinical features, survivals and patterns of mutual cooperativeness or exclusivity among cytogenetic and molecular genetics in large cohorts of patients, it’s clearly shown that the majority of AML cases can be classified into a number of biologically and prognostically distinct subgroups [23]. The 2017 ELN risk classification also works well in AML patients in Taiwan. (Fig. 3) It is suggested that patients with adverse-risk AML should be treated more aggressively to improve their survival.

**Table 2:** Functional categories of genes that are commonly mutated in acute myeloid leukemia (AML)

Functional category	Gene members	Role in AML Leukemogenesis
Myeloid transcription-factor genes	Transcription factor fusions by chromosomal rearrangements, such as t(8;21)(q22;q22); *RUNX1*-*RUNX1T1* and inv(16)(p13.1q22) or t(16;16)(p13.1;q22); CBFB-MYH11 *GATA2, RUNX1* and *CEBPA*	Transcriptional deregulation and impaired hematopoietic differentiation.
Nucleophosmin (*NPM1*) gene	*NPM1*	Aberrant cytoplasmic localization of NPM1 and its interacting proteins
Tumor suppressor genes	*TP53, WT1, PHF6*	Transcriptional deregulation and impaired degradation via the negative regulator (MDM2 and PTEN oncogenes)
Signaling genes	*FLT3, KIT, PTPN11, RAS*	Proliferative advantage through the RAS-RAF, JAK-STAT, and PI3K-AKT signaling pathways
DNA methylation	*DNMT3A, TET2, IDH1, IDH2*	Deregulation of DNA methylation and oncometabolite production
Chromatin modifier	*ASXL1, EZH2* and *KMT2A*	Deregulation of chromatin modification and impairment of methyltransferases function
Cohesin complex	*STAG1, STAG2, RAD21, SMC1A, SMC3,*	Impairment of accurate chromosome segregation and transcriptional regulation
Splicing factors	*SRSF2, SF3B1, U2AF1, ZRSR2*	Deregulated RNA processing and aberrant splicing patterns

**Table 3:** Risk stratification of AML according To 2017 ELN recommendations [24]

Risk profiles	Subgroups
**Favorable**	t(8;21)(q22;q22.1); *RUNX1-RUNX1T1*
	inv (16)(p13.1q22) or t(16;16)(p13.1;q22); *CBFB-MYH11*
	Mutated *NPM1* without *FLT3*-ITD
	Mutated *NPM1* with *FLT3*-ITD^low^
	Biallelic mutated *CEBPA*
**Intermediate**	Mutated *NPM1* and *FLT3*-ITD^high^
	Wild-type *NPM1* without *FLT3*-ITD
	Wild-type *NPM1* with *FLT3*-ITD^low^
	t(9;11)(p21.3;q23.3); *MLLT3-KMT2A*
	Cytogenetic abnormalities not classified
**Adverse**	t(6;9)(p23;q34.1); *DEK-NUP214*
	t(v;11q23.3); *KMT2A* rearranged
	t(9;22)(q34.1;q11.2); *BCR-ABL1*
	inv (3)(q21.3q26.2) or t(3;3)(q21.3;q26.2); *GATA2,MECOM(EVI1)*
	Complex karyotype, monosomal karyotype
	-5 or del(5q); −7; −17/abn(17p)
	Wild-type *NPM1* and *FLT3*-ITD^high^
	Mutated *RUNX1*
	Mutated *ASXL1*
	Mutated *TP53*

Low, low allelic ratio (< 0.5); high, high allelic ratio (≥0.5)

### Relevant genetic markers in AML

#### *FLT3 mutations*

FMS-like tyrosine kinase 3 gene (*FLT3*), located on chromosome 13q12, encodes a receptor tyrosine kinase that plays a major role in the regulation of hematopoiesis [26, 27]. There are two types of *FLT3* mutations; internal tandem duplication (ITD) of the FLT3 juxtamembrane domain, which are gain-of- function mutations [28, 29], and tyrosine kinase domain (TKD) point mutations, which occur mainly at codon 835 or 836 within the activation loop of the second kinase domain [30, 31]. The FLT3 mutant protein constitutively activates the cascade of FLT3 signaling in the absence of FLT3 ligand promoting cell proliferation and decreased apoptosis [32–34].

Mutations of *FLT3* are detected in approximately 25–30% of newly diagnosed AML patients as either ITD (20%) or point mutations in TKD (5–10%) [35–37]. Of note, the patients with *FLT3*-ITD have shorter disease-free survival (DFS), higher relapse rate and poorer OS [37–39], particularly among patients with high *FLT3*-ITD allelic ratio [40] or absence of *NPM1* mutation [39, 41, 42]. Besides, the insertion site and ITD length of FLT3 as well as concomitant mutations also appear to influence the prognosis [40, 43, 44]. For these reason, patients with *FLT3*/ITD are frequently referred for early allogeneic HSCT in first CR. Accordingly, the ELN and the National Comprehensive Cancer Center Consensus panels designated *FLT3*/ITD with high allelic ratio as an unfavorable prognostic subgroup [24, 45]. On the other hand, the prognostic impact of *FLT3*-TKD is not well defined [46, 47]. Up to one third of AML patients with *FLT3*-ITD or *FLT3*-TKD would lose the mutation at relapse whereas acquisition of novel *FLT3* mutations was detected in 20% patients at disease progression [48–50]. It is clinically significant to recheck *FLT3*-ITD or *FLT3*-TKD status at all subsequent treatment decision points in every patient regardless the *FLT3* status at diagnosis [51].

#### *KIT* mutations

KIT, known as stem cell factor receptor (cluster of differentiation 117, CD117), belongs to type III receptor tyrosine kinase family and is involved in the regulation of survival and proliferation of hematopoietic progenitor cells [52, 53]. KIT is highly expressed in the majority of leukemic blasts [54, 55], and *KIT* mutations, most commonly affecting exons 8 and 17 (especially D816 mutations), are identified in approximately 25% of AML with core binding factor (CBF) rearrangements [56–59], but infrequently found in other AML types [20]. The prognostic impact of *KIT* mutations in AML is controversial. Recently, the targeted high-throughput sequencing in 331 patients with t(8;21), which leads to CBF rearrangement, showed that *KIT* mutation, especially with higher allele burden, was independently associated with increased relapse rate and reduced OS.

#### *TP53* mutations

Somatic mutation of the tumor suppressor gene *TP53*, located in 17p13, is frequently detected in patients with therapy-related AML [60, 61] or AML with complex karyotype or monosomal karyotype (53–73%) [62–65], in contrast to 7–8% in de novo AML patients [19, 65]. In general, *TP53* mutations independently predict lower CR rate, higher relapse rate, shorter event-free survival (EFS) and OS.

#### *RAS* mutations

RAS proteins (HRAS, KRAS and NRAS), which are activated by cytokine receptors in response to ligand stimulation, control proliferation and survival of hematopoietic progenitors [66–69]. Activated *RAS* mutations are mostly single nucleotide substitutions in codon 12, 13 or 61 [70–73]. *NRAS* and *KRAS* mutations are found in 8–12% and 2–5% of AML patients, respectively. The prognostic relevance of *RAS* mutations in AML remains controversial. Higher dose of cytarabine was reported to decrease the relapse rate in *RAS*-mutated AML patients [74].

### Mutations of genes involving in epigenetic modifications

Deregulation of epigenetic modifiers, including alterations in DNA methylation, DNA hydroxymethylation and histone modifications, such as methylation, acetylation, phosphorylation, sumoylation and ubiquitination etc., is now recognized as an important mechanism in the pathogenesis of AML [75]. Somatic mutations in genes regulating epigenetic modifications, such as *IDH, TET2*, *DNMT3A, ASXL1*, *EZH2* and *KMT2A* are frequently detected in patients with AML, especially those with intermediate-risk cytogenetics [19, 76–80]. The epigenetic alterations usually present as the early pre-leukemic events [81–83] which cooperate with other genetic alterations in the development of AML. Mutations in *ASXL1*, *TET2,* and *DNMT3A* as well as *MLL* rearrangements confer poor prognosis, whereas *IDH* and *EZH2* mutations may bear clinical significance [75]. The treatment of choice for patients with epigenetic mutations is still under exploration. Induction chemotherapy with high-dose daunorubicin, as compared with standard-dose daunorubicin, improves OS among patients with *DNMT3A* mutations or *MLL* translocations [41]. Further, retrospective studies suggested that allogeneic HSCT in first CR can overcome the poor prognosis of patients with *MLL* rearrangements [84–86], *DNMT3A* mutations [87, 88], *ASXL1* mutations [89], and *IDH* mutations [90]. Nevertheless, prospective studies are warrant to clarify the point. Here we will specifically focus on *IDH*, *MLL*, and *EZH2* mutations in which targeted agents are either available in clinical use or under investigation.

#### IDH mutations

*IDH1* and *IDH2* genes encode two isoforms of isocitrate dehydrogenase, which catalyzes the oxidative decarboxylation of isocitrate to α-ketoglutarate (α-KG) [91]. Mutant IDH proteins convert α-KG to 2-hydroxyglutarate (2-HG), an onco-metabolite that contributes to tumor growth or malignant transformation [92, 93]. *IDH* mutations impair TET2-mediated hydroxymethylation of cytosine through reduced production of α-KG, a cofactor of TET2, thus result in global DNA hypermethylation [94]. Mutations in *IDH1* occur in 7.8–16% of patients with cytogenetically normal (CN) AML (CN-AML), and *IDH2* mutations, in 10–19% of those with CN-AML [75, 76, 78]. Both are less frequently detected in cytogenetically abnormal AML. Occasionally, *IDH* mutations can be detected in healthy older individuals with age-related clonal hematopoiesis, suggesting *IDH* mutations occur early in leukemogenesis [95]. The impact of *IDH* mutations on prognosis remains to be elucidated.

#### MLL rearrangements

The mixed lineage leukemia gene (*MLL*), also called Histone-lysine N-methyltransferase 2A (*KMT2A*), is located on chromosome 11q23 which encodes a DNA-binding protein that methylates histone H3 lysine 4 position (H3K4) and positively regulates Hox gene expression [96]. The incidence of *MLL* fusion and partial tandem duplication (*MLL/*PTD) in AML is approximately 5–12% and 5–6%, respectively [75] and the presence of *MLL* rearrangements usually predict aggressive course and poor outcome.

#### EZH2 mutations

Enhancer of Zest Homologue 2 gene (*EZH2*), located at chromosome 7q, is a H3K27 methyltransferase that catalyzes the Polycomb Repressive Complex 2 (PRC2) [97]. Mutations in *EZH2* exert context-specific and sometimes opposing effects on tumorigenesis. Oncogenic gain-of-function mutations are found in patients with lymphoid malignancies [98]; in contrast, loss-of-function *EZH2* mutations at diverse sites were detected in myeloid neoplasms [99, 100], including AML (0–2%) [75]. Further, derepression of stage-specific gene profiles induces polymorphic and contradictory phenotypes of EZH2 protein in different phases of AML [101, 102]. During AML maintenance, wild-type EZH2 exerts an oncogenic function as a facilitator of disease that may be therapeutically targeted. In contrast, EZH2 acts as a tumor suppressor during leukemia induction. The findings offer a potentially attractive therapeutic approach in AML with *EZH2* mutations and the EZH2 inhibitor is currently under development or early phase trials.

#### Splicing factor mutations

RNA splicing, a crucial post-transcription process, plays an important role in gene regulation and increases genomic diversity [103]. However, aberrant splicing pathologically drives the initiation and progression of cancers, including hematologic malignancies. Mutations of the splicing factor (SF) genes occur most frequently in *SRSF2, U2AF1, SF3B1* and *ZRSR2* [104]. The reported incidence of SF mutations in AML varied from 4.5 to 12.5% [19, 105–107]. Presence of SF mutations predict lower CR rate and shorter DFS and OS [107]. The discovery of somatic mutations in the spliceosome and/or aberrant splicing in cancers has prompted interest to novel therapeutic approaches by targeting splicing catalysis, splicing regulatory proteins, and individual key altered splicing events [108, 109].

### Targeted agents

Annotation of the mutational landscape in AML has greatly facilitated the development of targeted therapy. The breakthrough discovery of all-trans retinoic acid and arsenic trioxide in the treatment of acute promyelocytic leukemia [110], a specific subtype of AML, and tyrosine kinase inhibitor in chromic myeloid leukemia [111] has encouraged the searching for more novel targeted therapy in AML. In the past 3 years, the U.S. Food and Drug Administration (FDA) approved a total of eight drugs for AML; some specifically target certain gene mutations, biological pathways, or surface antigen. Based on the patients recruited in the clinical trials, most agents are approved at this moment for older patients or those with comorbidities, in whom the treatment choice is limited and the clinical outcome is very poor. It is expected that some of them may also be beneficial for younger and fit patients, but further clinical trials targeting this group of patients are needed to clarify it.

### FLT3 inhibitor

Given the adverse prognostic impact of *FLT3*-ITD and high frequency of *FLT3* mutations in de novo AML, *FLT3* mutations are attractive targets for therapy. Several FLT3 inhibitors are now available for clinical use, while more are under development in preclinical and clinical studies. FLT3 inhibitors can be classified into first and second generation inhibitors based on the potency and target specificity of the drugs. First generation FLT3 inhibitors, such as sunitinib, sorafenib, lestaurtinib and midostaurin, are relatively nonspecific multi-kinase inhibitors and generally have higher toxicities, while second generation inhibitors, such as quizartinib, crenolanib, and gilteritinib, are more selective and potent FLT3 inhibitors and have fewer toxicities. FLT3 inhibitors can be further classified into type I and II inhibitors based on the mechanism of interaction with the receptor. Type I inhibitors are effective for both *FLT3*-ITD and *FLT3*-TKD, while type II inhibitors, for *FLT3*-ITD only [35].

Midostaurin (PKC412) is the first FLT3 inhibitor approved by the U.S. as well as Taiwan FDA for the treatment of newly-diagnosed *FLT*3-mutated AML patients based on its effect on improving OS when combined with traditional chemotherapy [112, 113]. It is Type I inhibitor and effective regardless of types of *FLT3* mutations (ITD or TKD) or the allelic ratio of *FLT3*-ITD. Recently, maintenance of sorafenib, another first generation inhibitor, following allogeneic HSCT has shown encouraging results in *FLT3*-mutated AML by reducing the post-transplant relapse rate [114, 115].

As for second generation FLT3 inhibitors, Crenolanib and gilteritinib are type I inhibitors, whereas quizartinib is type II inhibitor [116]. Gilteritinib has single-agent activity in *FLT3-*mutated AML and was approved by the U.S. FDA in November 2018 for treating adult patients who have relapsed or refractory (R/R) *FLT3*-mutated AML based on safety data and an interim analysis of the response rate in the ADMIRAL trial (NCT02421939) [117]. The final results showed that the median OS and event-free survival (EFS) in the gilteritinib group was significantly longer than that in the chemotherapy group. The clinical trials to investigate its use as frontline treatment or maintenance in AML patients with *FLT3* mutations are undergoing. Quizartinib (AC220) was shown effective as single agent in R/R *FLT3*/ITD patients with improving OS compared to chemotherapy. However, Quizartinib is only approved in this setting in Japan, but not in the USA and European Union (European Medicines Agency, EMA) due to marginal survival benefits and safety concerns. A number of other novel FLT3 inhibitors, such as tandutinib, crenolanib, cabozantinib, etc., are currently under development or in clinical trials [118].

Collectively, FLT3 inhibitors has emerged as an important part of therapy for *FLT3*-mutated patients in both frontline and R/R status. Much is still to be learned about how to advance the use of FLT3 inhibitors in fit or frail patients (such as novel combinations), overcome the primary and secondary acquired resistance, and manage the adverse effects, especially in maintenance therapy.

### KIT inhibitor

*KIT* mutations occur frequently in CBF AML and may confer poorer prognosis in this group of patients. Since dasatinib is a potent oral multi-kinase inhibitor with strong activity on KIT oncoprotein, it has the potential to target this molecular aberration in AML patients. A phase 2, open-label, multicenter trial (CALGB10801) showed that chemotherapy plus dasatinib was well tolerated without any unexpected or dose-limiting toxicities [119]. It provided excellent outcomes (90% CR rate and 77% OS at 3-year) to both younger and older patients with *KIT* mutations, supporting further large-scale, prospective randomized phase 3 trials to evaluate KIT inhibitors in combination with cytotoxic chemotherapy in the treatment of *KIT-*mutated CBF AML.

### TP53 inhibitor

A growing number of small low-molecular-weight compounds including PRIMA-1 and the PRIMA-1 analog APR-246 have been developed to restore tumor suppressor function to mutant p53 [120–123]. In a preliminary analysis of 45 *TP53*-mutated patients with myelodysplastic syndrome (MDS), a pre-leukemia state, and oligoblastic AML in a phase Ib/II combination study (APR-246 and azacitidine; NCT03072043) [124], the overall response rate (ORR) was 87% with 53% CR. The median time to response was 2.1 months (range, 0.1–5.4) and the median duration of response was 6.5 months with a median follow-up of 10.5 months. The randomized phase 3 study of APR-246 and AZA versus AZA alone in *TP53-mutated* MDS is ongoing (NCT03745716)**.**

### RAS inhibitor

Since farnesylation is the primary translational modification essential for the transforming activity of RAS oncoprotein, attempts to target RAS with farnesylation inhibitors have been developed since 2000 [69, 125]. Although preclinical activity was observed in *RAS*-mutant cell lines and animal models, clinical activity of farnesylation inhibitors in AML patients has been largely unsuccessful and disappointing [126–128]. Clinical trials targeting mitogen-activated protein kinase (MAPK) signaling in *NRAS*-mutated leukemia with MAP-ERK kinase (MEK) inhibitors are ongoing; the response is only minimal though MEK inhibitors are generally well tolerated [129, 130]. Further studies are required to explore other small-molecular inhibitors, select suitable patient cohort and investigate synergistic combination therapies.

### IDH inhibitor

Enasidenib (AG-221), first *IDH* mutation-specific inhibitor, suppresses 2-HG production and induces cellular differentiation in primary human *IDH2*-mutated AML cells and xenograft mouse models [131]. In the interim analysis of the landmark first-in-human phase I/II trial for enasidenib (NCT01915498) [132], the ORR was 38.5%, including 20.2% CR in 109 adult R/R patients with *IDH2*-mutated AML receiving 100 mg daily. The median time to CR was 3.7 months and the duration of response in patients who attained CR was 8.8 months (range, 6.4-not reached). Accordingly, the U.S. FDA approved enasidenib in August 2017 for the treatment of *IDH2*-mutated R/R AML. The final analysis of this trial (*n* = 345) showed 46% attained their best response by cycle 4 and 80%, by cycle 6 among responding patients, implying failure to obtain early response with enasidenib does not necessarily indicate treatment failure and the importance of continuing enasidenib therapy for at least 5–6 cycles [133]. The clearance of *IDH2-*mutant clones was associated with achievement of CR. Clinical trials including enasidenib for R/R AML (phase 3, NCT02577406), newly diagnosed AML (NCT02632708) and post allogeneic HSCT maintenance (NCT03515512), and in combination with azacitidine for R/R AML (NCT03683433), etc. are ongoing.

Ivosidenib (AG-120) is a highly selective inhibitor for *IDH1* mutants. It lowers 2-HG in tumor models and enhances differentiation of primary AML samples [134]. Ivosidenib monotherapy was associated with durable remissions in 179 patients with *IDH1*-mutated, R/R AML in a phase 1 dose-escalation and dose-expansion study (NCT02074839) [135]. The ORR was 41.6%, including 30.4% CR/CR with partial hematologic recovery (CRh); 21% of patients who had a CR or CRh had no residual detectable *IDH1* mutations on digital polymerase-chain-reaction assay. The median time to CR/CRh was 2.7 months (range, 0.9–5.6) and the duration of response in these patients was 8.2 months (range, 5.5–12). In a trial of ivosidenib for patients with newly-diagnosed *IDH1*-mutated AML, the CR/CRh rate was 42% and the median duration of response was not reached in 34 patients who received ivosidenib 500 mg once daily [136]. Based on these findings, the U.S FDA approved ivosidenib in both the frontline and salvage treatment of *IDH1*-mutated AML.

### Epigenetic therapies

#### Hypomethylating agents (HMA)

The hypomethylating agents (HMA), azacytidine and decitabine, have long been known for their effects in AML patients with low blast percentages (20–30%) [137, 138]. However, their effects in AML patients with higher blast percentages are not impressive, and efforts now are focused on the combination of the drug with other novel agents. Guadecitabine (SGI-110), a next-generation HMA, is a dinucleotide of decitabine and deoxyguanosine resulting in slow release of decitabine and prolonged in vivo half-life; therefore, guadecitabine is potentially more effective and less toxic than its parent drug [139]. Unfortunately, phase III ASTRAL-1 study failed to meet the primary endpoint of a statistical difference in CR and OS between guadecitabine and control arm [140]. Nevertheless, a benefit was observed in subgroup of patients who received 4 or more cycles, indicating that treatment duration is crucial to response. The phase III QUAZAR AML-001 study (NCT01757535) demonstrated that maintenance with CC-486, an oral formulation of 5-azacitidine, resulted in significant improvements in OS and RFS, compared with placebo [141]. CC-486 is the first HMA used in the maintenance setting to improve clinical outcome in patients with AML after achieving remission following induction chemotherapy, with or without consolidation.

#### DOT1L inhibitor

The histone 3 lysine 79 (H3K79) methyltransferase disruptor of telomeric silencing-1 like (DOT1L) is proposed to play a role in the development of leukemia in patients with *MLL* translocations [142, 143]. Pharmacological inhibition of DOT1L enzymatic activity has been of interest for the treatment of *MLL*-rearranged leukemias [144]. The DOT1L inhibitor Pinometostat (EPZ − 5676) exhibited modest clinical activity in a phase I study, which paves a road for further exploration of combination therapies in leukemia [145].

#### Bromodomain and extra-terminal (BET) protein inhibitors

Bromodomain and extra-terminal (BET) proteins bind acetylated lysine residues on histone tail to facilitate transcriptional activation [146]. BET proteins are involved in aberrant chromatin states in AML through MYC upregulation [147]. BET inhibitors, such as JQ1 and OTX015 (MK-8628) showed efficacy in cell lines, mouse models and ex vivo patient samples of *MLL*-fused, *NPM1*-, *FLT3*-, or *IDH2*-mutated leukemias [148]. So far single-agent activity is modest in R/R AML [149] and the early phase studies of several BET inhibitors are ongoing.

#### Lysine-specific demethylase 1 (LSD1) inhibitors

Lysine-specific demethylase 1 (LSD1), an enzyme responsible for the demethylation of H3K4 and H3K9, is an essential regulator to sustain the oncogenic potential of leukemic stem cells [150]. Several LSD1 inhibitors have shown in vitro anti-leukemic activity but also striking hematologic toxicity in mouse models [150, 151]. Ladademstat (ORY-1001), a highly potent and selective LSD1 inhibitor, induced blast differentiation and reduction of leukemic stem cell capacity in AML [152], and exhibited potent synergy with standard-of-care drugs and selective epigenetic inhibitors [152].

#### Histone deacetylase inhibitors

Several inhibitors of histone deacetylases (HDAC), such as panobinostat and vorinostat, have been developed [153, 154]. However, monotherapy with HDAC inhibitors only showed modest effect in AML [155].

### BCL2 inhibition

The B-cell leukemia/lymphoma-2 (BCL-2), a key regulator of the mitochondrial apoptotic pathway, supports cell survival by suppressing programmed cell death [156, 157]. BCL2 is aberrantly overexpressed in AML blasts, specifically in leukemic stem cells [158], and enhanced BCL-2 expression mediates chemotherapy resistance [159, 160]. Venetoclax is a highly selective BH3 mimetic agent showing potent BCL-2 inhibition. Venetoclax-based therapy for heavily pretreated patients with R/R AML showed fair activity with 19–22.5% of patients achieving CR or CR with incomplete hematologic recovery (CRi) [161, 162]. Impressively, in phase Ib/II clinical trials, venetoclax in combination with low-dose cytarabine (LDAC) or HMAs in treatment-naive patients showed very exciting results; rapid and deep response could be seen in 54–67% of patients aged 75 years or older or those with comorbidities that precluded intensive chemotherapy [163, 164]. The response to venetoclax-based therapy is mostly observed within 1–2 cycles and the median survival is not reached for patients obtaining CR/CRi. The results served as the basis for accelerated approval by the U.S. FDA in Nov 2018 and herald a new era of AML therapy that largely avoids traditional cytotoxic agents in unfit patients.

The phase III trials comparing venetoclax and azacitidine to azacitidine alone (NCT02993523) and venetoclax and LDAC to LDAC alone (NCT03069352) are ongoing to confirm the clinical benefits. Besides, venetoclax is also tested to combine with other targeted agents, such as IDH inhibitors in *IDH*-mutated patients or FLT3 inhibitors in *FLT3*-mutated patients, to evaluate if such combinations can enhance anti-leukemic efficacy.

### Other agents

In addition to the targeted therapies mentioned above, the most promising agents for non-mutation-targeted novel agents approved by the U.S. FDA include CPX-351 (Vyxeos), gemtuzumab ozogamicin (Mylotarg), and glasdegib (Daurismo). CPX-351 is a dual drug liposomal encapsulation of daunorubicin and cytarabine which is approved for the treatment of therapy-related AML and AML with myelodysplasia-related change [165]. Gemtuzumab ozogamicin (Mylotarg) is a humanized anti-CD33 monoclonal antibody linked to calicheamicin which is approved for newly-diagnosed and R/R CD33+ AML [166]. Glasdegib (Daurismo) is the first Hedgehog pathway inhibitor. It is approved in combination with low-dose cytarabine for newly diagnosed AML aged 75 or more or those who have comorbidities that preclude use of intensive induction chemotherapy [167].

## Conclusion

Recent advances in genomics techniques have unraveled the molecular heterogeneity of AML leukemogenesis and further help refine risk stratification and prognostication. Patients with adverse-risk AML require more aggressive treatment including allogeneic HSCT in first CR and possibly novel targeted agents, to improve the prognosis. However, the complex pattern of cooperativity and mutual exclusivity among different mutations remain a clinical challenging. Since 2017 there has been an explosion of newly approved treatment options to tailor personalized treatment for AML. Each of these targeted therapies has unique treatment timing, dosing, efficacy, and adverse effects and appropriate management is crucial to the success of treatment. Further combinations of molecularly targeted therapies and standard cytotoxic chemotherapy or other novel agents to enhance efficacy are still under investigation. We believe it is clinically relevant to comprehensively elucidate the molecular signatures to better characterize the AML biology, precisely predict prognosis and tailor treatment strategies with targeted agents.

## Data Availability

Non applicable.
